# The Different Microbial Etiology of Prosthetic Joint Infections according to Route of Acquisition and Time after Prosthesis Implantation, Including the Role of Multidrug-Resistant Organisms

**DOI:** 10.3390/jcm8050673

**Published:** 2019-05-13

**Authors:** Natividad Benito, Isabel Mur, Alba Ribera, Alex Soriano, Dolors Rodríguez-Pardo, Luisa Sorlí, Javier Cobo, Marta Fernández-Sampedro, María Dolores del Toro, Laura Guío, Julia Praena, Alberto Bahamonde, Melchor Riera, Jaime Esteban, Josu Mirena Baraia-Etxaburu, Jesús Martínez-Alvarez, Alfredo Jover-Sáenz, Carlos Dueñas, Antonio Ramos, Beatriz Sobrino, Gorane Euba, Laura Morata, Carles Pigrau, Juan P. Horcajada, Pere Coll, Xavier Crusi, Javier Ariza, on behalf of the REIPI (Spanish Network for Research in Infectious Disease) Group for the Study of Prosthetic Joint Infections/GEIO (Group for the Study of Osteoarticular Infections), SEIMC (Spanish Society of Infectious Diseases and Clinical Microbiology)

**Affiliations:** 1 Infectious Diseases Unit, Hospital de la Santa Creu i Sant Pau - Institut d’Investigació Biomèdica Sant Pau, 08025 Barcelona, Spain; imur@santpau.cat; 2 Department of Medicine, Universitat Autònoma de Barcelona, 08193 Barcelona, Spain; pcoll@santpau.cat; 3 Department of Infectious Diseases, Hospital Universitari Bellvitge, 08097 Barcelona, Spain; albaribera@gmail.com (A.R.); geubuga@gmail.com (G.E.); jariza@bellvitgehospital.cat (J.A.); 4 Department of Infectious Diseases, Hospital Clínic Universitari, 08036 Barcelona, Spain; asoriano@clinic.cat (A.S.); lmorata@clinic.cat (L.M.); 5 Department of Infectious Diseases, Hospital Universitari Vall d’Hebron, 08035 Barcelona, Spain; dolorodriguez@vhebron.net (D.R.-P.); cpigrau@vhebron.net (C.P.); 6 Department of Infectious Diseases, Parc de Salut Mar, 08003 Barcelona, Spain; lsorli@parcdesalutmar.cat (L.S.); jhorcajada@parcdesalutmar.cat (J.P.H.); 7 Department of Infectious Diseases, Hospital Universitario Ramón y Cajal – IRYCIS, 28034 Madrid, Spain; javier.cobo@salud.madrid.org; 8 Department of Infectious Diseases, Hospital Universitari Valdecilla, 39008 Santander, Spain; martafersam@yahoo.es; 9 Department of Infectious Diseases, Clinical Microbiology and Preventive Medicine, Hospital Universitario Virgen Macarena, 41009 Sevilla, Spain; mdeltoro@us.es; 10 Department of Infectious Diseases, Hospital Universitario Cruces, 48903 Bilbao, Spain; laura.guiocarrion@osakidetza.eus; 11 Department of Infectious Diseases, Clinical Microbiology and Preventive Medicine, Hospital Universitario Virgen del Rocío, 41013 Sevilla, Spain; juliapraena@gmail.com; 12 Department of Internal Medicine-Infectious Diseases, Hospital Universitario del Bierzo, 24404 León, Spain; med007783@me.com; 13 Infectious Diseases Unit, Department of Internal Medicine, Hospital Universitario Son Espases, 07120 Palma de Mallorca, Spain; melchor.riera@ssib.es; 14 Department of Clinical Microbiology, IIS-Fundación Jiménez Díaz, 28040 Madrid, Spain; jestebanmoreno@gmail.com; 15 Department of Infectious Diseases, Hospital Universitario de Basurto, 48013 Bilbao, Spain; josumirena.baraia-etxaburuartexe@osakidetza.eus; 16 Department of Orthopedic and Traumatology, Hospital Universitario Central de Asturias, 33011 Oviedo, Spain; j.martinez-alvarez@hotmail.com; 17 Unit of Nosocomial Infection, Hospital Universitari Arnau de Vilanova, 25198 Lleida, Spain; ajover.lleida.ics@gencat.cat; 18 Department of Internal Medicine, Hospital Clínico Universitario de Valladolid, 47003 Valladolid, Spain; carlos.duenas@hotmail.com; 19 Infectious Diseases Unit, Department of Internal Medicine, Hospital Universitario Puerta de Hierro, 28222 Madrid, Spain; aramos220@gmail.com; 20 Department of Infectious Diseases, Hospital Regional Universitario Málaga, 29010 Málaga, Spain; bea_sobrino@yahoo.es; 21 Department of Clinical Microbiology, Hospital de la Santa Creu i Sant Pau - Institut d’Investigació Biomèdica Sant Pau, 08025 Barcelona, Spain; 22 Department of Orthopedic and Traumatology, Hospital de la Santa Creu i Sant Pau - Institut d’Investigació Biomèdica Sant Pau, 08025 Barcelona, Spain; xcrusi@santpau.cat

**Keywords:** prosthetic joint infections, microbial etiology, classification schemes for prosthetic joint infections, antimicrobial empirical treatment, multidrug-resistant organisms

## Abstract

The aim of our study was to characterize the etiology of prosthetic joint infections (PJIs)—including multidrug-resistant organisms (MDRO)—by category of infection. A multicenter study of 2544 patients with PJIs was performed. We analyzed the causative microorganisms according to the Tsukayama’s scheme (early postoperative, late chronic, and acute hematogenous infections (EPI, LCI, AHI) and “positive intraoperative cultures” (PIC)). Non-hematogenous PJIs were also evaluated according to time since surgery: <1 month, 2–3 months, 4–12 months, >12 months. AHIs were mostly caused by *Staphylococcus aureus* (39.2%) and streptococci (30.2%). EPIs were characterized by a preponderance of virulent microorganisms (*S. aureus,* Gram-negative bacilli (GNB), enterococci), MDROs (24%) and polymicrobial infections (27.4%). Conversely, coagulase-negative staphylococci (CoNS) and *Cutibacterium* species were predominant in LCIs (54.5% and 6.1%, respectively) and PICs (57.1% and 15.1%). The percentage of MDROs isolated in EPIs was more than three times the percentage isolated in LCIs (7.8%) and more than twice the proportion found in AHI (10.9%). There was a significant decreasing linear trend over the four time intervals post-surgery for virulent microorganisms, MDROs, and polymicrobial infections, and a rising trend for CoNS, streptococci and *Cutibacterium* spp. The observed differences have important implications for the empirical antimicrobial treatment of PJIs.

## 1. Introduction

While the risk of prosthetic infection in patients undergoing joint replacement could be considered low (hips: 0.2–1.5%, knees: 0.4–1.5%, shoulders: 0.8–2%), the high frequency of these procedures converts the combination of low risks into a substantial burden of infection [1]. Prosthetic joint infection (PJI) is a devastating complication associated with major patient morbidity and high healthcare and societal costs (recent estimated costs of 20,000–40,000 dollars per infection) [1].

Biofilm formation when microorganisms attach to the surface of prosthetic devices plays a crucial role in the pathogenesis of PJI [2]. This poses a challenge for the diagnosis of biofilm-embedded microorganisms, and antimicrobial therapy in biofilm-associated pathogens is of limited efficacy [3].

Antimicrobial therapy combined with surgery is required to cure PJIs [4,5,6,7]. A common management approach is to start broad-spectrum antimicrobial agents after obtaining intraoperative samples for culture [8,9]. The importance of adequate initial empirical antimicrobial therapy in the outcome of infections is well-known [10] and seems to be critical in patients with PJIs treated with debridement, antibiotics, and implant retention [11]. Vancomycin combined with a broad-spectrum beta-lactam such as piperacillin-tazobactam has been recently studied as an initial treatment [12,13] but has been associated with a high rate of adverse effects [13]. After the pathogen has been identified and antimicrobial susceptibility results are available, the most effective narrow-spectrum antibiotic regimen is selected for continuation of therapy [4,5,6,7,8,10]. Nevertheless, a significant number of patients (5–35%) have negative cultures [8]. In this situation, empirical antimicrobial therapy is even more important, but more difficult to decide on, taking into consideration that the patient will have to receive it for the several weeks to months that it takes to cure a PJI [4,8].

Knowledge of the microbiological spectrum of PJIs is essential for guiding empirical antibiotic therapy. There are, however, no specific recommendations for the most appropriate empirical treatment for PJIs. We previously characterized the microbial etiology of PJI in a large cohort of patients [14], but the causative microorganisms can vary significantly, depending on the infection route and the time interval between index surgery and onset of symptoms, which can help guide empirical treatment [4,8,12,15]. These differences may include the involvement of multidrug-resistant microorganisms (MDRO), although this aspect has not been previously studied. There are several useful classifications of PJI in different categories, namely based on the mode of acquisition and/or time from prosthesis implantation, but none of them are universally accepted [8,16]. A good deal of what is currently known about the microbial etiology of different categories of PJI is based on studies that are limited by small sample sizes [11,15,17,18,19,20,21,22,23,24,25,26,27,28,29,30,31] and describe single-center experiences [12,15,17,18,19,20,21,22,23,24,26,27,28,30,31,32]. Most focus on specific types of infection, [11,21,23,25,29] surgical strategies used during treatment [22,31], or include only infections occurring within a limited period of time after prosthesis implantation [30,33]. Consequently, the results do not adequately represent the percentages of the different microorganisms involved in different types of infection across the full range of PJIs

Our aim was to characterize the etiology of PJIs—including MDROs—according to the category of infection, in a large cohort of consecutive patients with PJIs. The results would enable tailoring empirical antimicrobial therapy to the clinical situation, offering coverage of the most likely microorganisms but narrowing the antimicrobial spectrum, which is crucial in antimicrobial stewardship program, and potentially reducing adverse effects. 

## 2. Methods

### 2.1. Setting, Study Design, and Patients

This was an ambi-directional observational study carried out at 19 hospitals in different areas of Spain. The study was performed within the framework of the Spanish Network for Research in Infectious Diseases (REIPI) (www.reipi.org) and included the participation of the Group for the Study of Osteoarticular Infections (GEIO) (https://seimc.org/grupos-de-estudio/geio) belonging to the Spanish Society of Clinical Microbiology and Infectious Diseases (SEIMC). The REIPI Group for the Study of PJIs and the GEIO form a multicenter collaborative group of infectious disease specialists, microbiologists, and orthopedic specialists across Spain, with extensive experience at orthopedic infection management and had previous joint publications [14,29,34,35,36]. 

All consecutive adult patients diagnosed with PJIs between 2003 and 2012 were included. Excluded were relapse episodes of infections that were first diagnosed before the study period.

### 2.2. Data Collection

This study was ambi-directional, with both prospective and retrospective data collection. Data were first acquired from the REIPI cohort of consecutive patients with PJI who were prospectively enrolled from 2003 through 2006. The cohort characteristics have been described elsewhere [29,34]. Apart from clarifications concerning key variables, no further data on this prospective cohort was requested. For the retrospective phase, data of patients who developed PJIs from 2007 through 2012 were retrospectively collected from REIPI and other hospitals that met the criteria for participation. The three criteria included hospitals with orthopedic surgery, the use of proper identification procedures to ensure the inclusion of all consecutive cases diagnosed at the hospital, and that ascertainment bias was minimized, and finally, most of the data needed to resolve queries were either available or easily accessible. A standard case report form designed specifically for this study was used at all sites to collect data. Data of patients with PJI were obtained from electronic databases used at most of the participating hospitals with prospectively collected information on patients with PJI, and from the patient’s medical records held at each hospital as required. Completed case report forms were sent to the coordinating center for data entry, or the site investigators entered the variables directly into the common electronic database. The coordinating center for this study was the Hospital de la Santa Creu i Sant Pau (Barcelona, Spain). The study was approved by the Institutional Review Board at the Hospital de la Santa Creu i Sant Pau before data collection started. All case report forms were reviewed at the coordinating center.

### 2.3. Clinical Data and Definitions

The following information was collected: Patient demographics and underlying conditions, characteristics of the arthroplasty, risk factors for MDROs, classification of the PJI, and microbiological diagnosis. All variables were predefined to ensure standardized data collection in participating hospitals. Individual patient data recorded included age and gender; comorbidities and immunosuppressive therapy; the patient’s American Society of Anesthesiologists (ASA) score before the surgical procedure (typically the arthroplasty implant) closest to the diagnosis of infection; previous exposure to antibiotics (≥7 days) or hospitalization in the previous 90 days (≥2 days); receipt of hemodialysis, intravenous therapy, wound care or specialized nursing care at home in the 30 days preceding the last surgical procedure or onset of hematogenous PJI; residence in a nursing home or long-term care facility. Information of the arthroplasty collected included: The reasons for implantation and the date performed, site, time from admission to implantation, primary or revision arthroplasty, cemented vs. uncemented, and the use of antibiotic-loaded bone cement. Date of diagnosis, classification of the PJI type, and the number of cultured samples and their results were also recorded. 

Cefazolin —sometimes cefuroxime, depending on the center, — was used as an antimicrobial prophylaxis in surgery. Vancomycin or teicoplanin was used for patients who were allergic to penicillin. Baseline comorbidities were quantified using the Charlson comorbidity score [37]. A diagnosis of PJI was established using the 2011 Musculoskeletal Infection Society definition [38]. The microbial etiology of PJI was established when the same organism (indistinguishable by common laboratory tests including genus and species identification or common antibiogram) was isolated in two or more periprosthetic cultures yielded [4]. When the diagnostic criteria for PJI were met, virulent microorganisms (such as *Staphylococcus aureus*) isolated in a single periprosthetic tissue/biopsy sample were also considered causative organisms [7]. MDRO was defined following Magiorakos et al. (acquired non-susceptibility to at least one agent in three or more specified antimicrobial categories) [39].

The Tsukayama scheme was used to classify PJIs. This scheme divides PJIs into four categories, based partly on presumed mode of infection and time since surgery: [15,40] a) Early postoperative infection (EPI): PJI diagnosed within one month of the index surgery (usually implantation of joint prosthesis, but also later procedures performed at the arthroplasty site); b) late chronic infection (LCI): PJIs with an insidious clinical course diagnosed >1 month after the index operation; both EPI and LCI are considered perioperatively acquired; c) acute hematogenous infection (AHI): PJI associated with documented or suspected antecedent bacteremia and characterized by acute onset of symptoms in the affected joint with the prosthesis; d) positive intraoperative culture (PIC): PJI diagnosed when at least two specimens, from a minimum of five obtained at the time of revision surgery, are positive after culture; infection was not clinically obvious or suspected at the time of the revision [15,40]. AHI and PIC can occur any time after surgery. EPI and AHI are acute PJIs that can be treated and potentially cured with debridement and antibiotics, without removal of the prosthesis. Another commonly used classification scheme based only on time since index surgery classifies PJIs as early (develops <3 months after surgery), delayed (3 to 12 or 24 months after surgery), or late (>12 or 24 months after surgery) [8,41]. This chronological framework was used to further analyse all cases of non-hematogenous PJI.

### 2.4. Statistical Analysis

Medians and interquartile ranges were used to summarize continuous variables, and absolute numbers and percentages of total samples for categorical variables. Statistical analyses were based on differences in the percentages of causative microorganisms/groups of organisms of PJI in the four categories of the Tsukayama scheme (EPI, LCI, AHI, PIC). The causative microorganisms of non-haematogenous PJIs diagnosed according to time since surgery (within the first month, in months 2/3, months 4–12, and more than 12 months after the index surgery), were also compared. These percentages were compared using the χ2 test or Fisher’s exact test as appropriate. To determine statistically significant linear trends in the proportions of infection caused by specific microorganisms/groups of organisms over time the Mantel-Haenszel χ2 test for trend was used. The data were analyzed using SPSS, version 22.0 (IBM SPSS, Chicago, IL, USA)

## 3. Results

Overall, 2524 episodes of PJI were diagnosed during the study period in 19 participating hospitals located in eight of the 17 administrative regions of Spain. All were university hospitals except for one. In 17 hospitals, they had more than 500 beds, and two had between 400 and 500 beds. 

The characteristics of the patients are outlined in Table 1. Most infections occurred in hip or knee arthroplasties; 77.2% affected primary arthroplasties. The most common reason for joint replacement was degenerative joint disease.

LCI was the most frequent type of infection, accounting for 47.4% (1178) of cases, followed by EPI (35.7%, 888), AHI (11.6%, 288) and PIC (5.3%).

A microbiological diagnosis was obtained in 2288 cases (90.6%) and significantly more frequently in EPI (94.5%, 839) and AHI (92%, 256) than in LCI (89.3%, 1052) (*p* < 0.001).

The causative microorganisms of PJI using Tsukayama’s classification are shown in Table 2 and Table 3. Overall, coagulase-negative staphylococci (CoNS) were the most common group of microorganisms involved in PJI (with *Staphylococcus epidermidis* as the most frequent species). They were more often isolated in chronic (>50% of cases) than in acute infections. Whereas CoNS represented almost 30% of EPIs, they were involved in less than 10% of AHI. *S. aureus* on the other hand, was the microorganism most often involved in acute infection and the leading causative species of EPI and AHI. Streptococci were significantly more common in AHI, while enterococci were more frequent in EPI than in other categories of PJI.

Aerobic Gram-negative bacilli (GNB), both *Enterobacteriaceae* and non-fermenting Gram-negative bacilli, were much more frequently involved in EPI than in the other types of infection. *Escherichia coli,* however, was isolated almost as frequently in AHI as in EPI. 

*Cutibacterium* (formerly *Propionibacterium*) spp. were more common in chronic than in acute PJI, but with a significantly higher proportion in the PIC group (15.1%) than in the LCI (6.1%).

Polymicrobial infections were much more frequent in the EPI category (27.4%) than in other types, and MDROs (both methicillin-resistant *S. aureus* (MRSA) and multidrug-resistant GNB, including extended-spectrum beta-lactamase (ESBL)-producing *Enterobacteriaceae*) were also much more commonly isolated in EPIs than in other categories of infection. Ciprofloxacin-resistant GNB were also more common in EPIs. 

With respect to non-haematogenous infections, a microbiological diagnosis was obtained for 94.5%, 92.7%, 92%, and 88.2% of cases in the first month, months 2/3, 4–12 and more than 12 months after index surgery, respectively, with a statistically significant decreasing linear trend (*p* < 0.001). With respect to PJIs diagnosed in the first month after surgery (EPI according to the Tsukayama classification) versus those diagnosed in the second or third month after surgery (also early infections according to other common classifications) [5,36], the former were significantly more often caused by *S. aureus*, enterococci, aerobic GNB (with more than twice the percentage of both *Enterobacteriaceae* and non-fermenting GNB than in months 2 and 3), MDROs (both MRSA and multidrug-resistant GNB, including ESBL-producing *Enterobacteriaceae*) and polymicrobial infections, and less often caused by CoNS, streptococci, and *Cutibacterium* spp. (Table 4). Furthermore, infections diagnosed more than three months after surgery were more frequently caused by CoNS, and less commonly by *Enterobacteriaceae* than those diagnosed in the first two to three months after surgery. No other differences between these four time intervals were observed. When the four periods of time after the index surgery were considered overall, a statistically significant decreasing linear trend was observed for infections caused by *S. aureus*, enterococci, *Enterobacteriaceae*, non-fermenting GNB (mainly *Pseudomonas* spp.), MDROs (both MRSA and multidrug-resistant GNB), and polymicrobial infections (*p* < 0.001 in each case) and a rising trend for infections caused by CoNS (*p* < 0.001), streptococci (*p* = 0.015) and anaerobic bacteria (mainly *Cutibacterium* spp.) (*p* < 0.001). 

**Figure 1:**
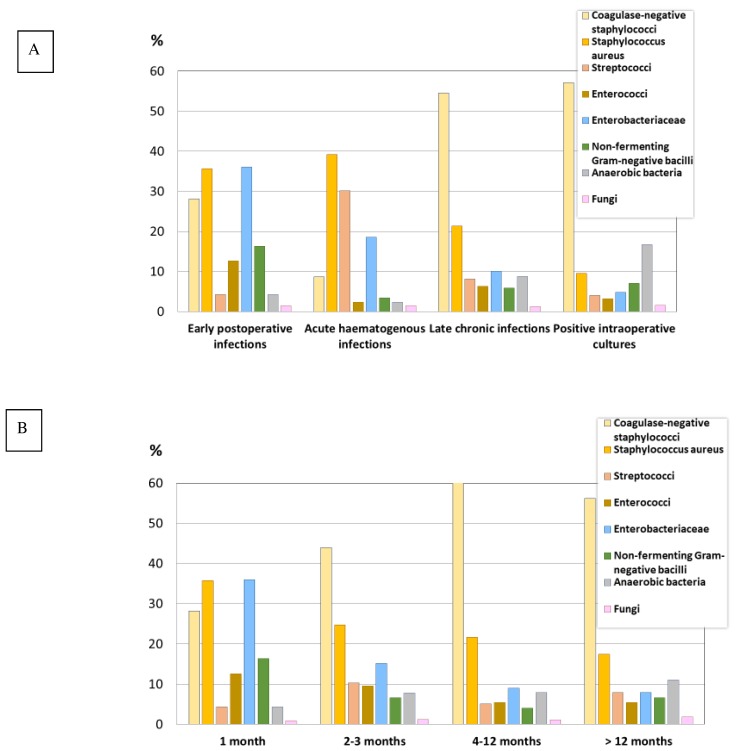
Main microorganisms or group of microorganisms involved in prosthetic joint infections according to Tsukayama’s classification (**A**), and in non-hematogenous prosthetic joint infection according to time since index surgery (**B**).

Figure 1 shows the main microorganisms/groups of organisms involved in each of the Tsukayama categories of PJI (A) and in non-haematogenous PJIs according to time since index surgery (B). 

## 4. Discussion

Empirical antimicrobial treatment is based on a diagnosis of infection without knowing the causative microorganism, while covering those most likely to be involved in particular clinical situations [42]. This multicenter study, which is, to our knowledge the largest series to have analyzed this question, found significant differences in the microbial etiology of different types of prosthetic joint infection. Our results show that the choice of empirical antibiotics for early postoperative infections (EPI) (<1 month after surgery) is the most challenging, since these infections are characterized by a preponderance of diverse and virulent pathogens, mainly *S. aureus, Enterobacteriaceae, Pseudomonas aeruginosa* and enterococci, MDROs, and polymicrobial infections. These microorganisms and groups of organisms were isolated progressively less frequently in non-hematogenous PJIs after the first month following index surgery, whereas a steady linear increase over time was observed for less virulent microorganisms, such as CoNS and *Cutibacterium* spp. (frequently considered commensals). Multidrug-resistant GNB were rarely encountered in non-EPI infections, and MRSA was isolated twice as frequently in EPIs as in LCIs. The pattern for AHI was different, with *S. aureus* and streptococci together accounting for almost 70% of infections. These results could help in the selection of an empirical antimicrobial therapy that is tailored to specific clinical situations, with coverage of the most likely microorganisms, but the narrowest possible antimicrobial spectrum.

As in other previous series, the most commonly cultured microorganisms in PJIs belonged to the CoNS group (with *S. epidermidis* the most common species) [38]. These bacteria are ubiquitous members of the human skin microbiome and lack aggressive virulence properties overall, but nevertheless account for the most device-associated infections, mainly because of their capacity for biofilm production [43]. These characteristics would explain our finding of CoNS as a common cause of perioperatively-acquired PJI, with clinical manifestations increasingly more common after the early postoperative period, but rarely a cause of AHI. In spite of significant differences between the CoNS species [43], we found that isolates of CoNS species behaved in a similar way, although it should be remembered that a substantial percentage of CoNS were not identified to the species level.

As in nearly all previous series, *S. aureus* was the most common bacterial species found as a cause of PJI (followed by *S. epidermidis)* [44,45]. *S. aureus* was the most frequently found causative pathogen of AHI, probably because of its high virulence. In previous studies, PJI following *S. aureus* bacteremia was observed in 30 to 40% of patients with prosthetic joints. Furthermore, non-hematogenous PJIs caused by *S. aureus* most often presented early [45]; our results showed a linear percentage decrease in *S. aureus* after the first month post-surgery. 

Overall, streptococci and enterococcus species cause only 9% and 8% of PJIs, respectively, and are more often involved in acute than chronic infections. However, while streptococci are found in a noteworthy 30% of AHI (the second most common cause of this type of infection after *S. aureus*), enterococci are more common in EPI, although only accounting for 13% of these infections, a similar percentage to those observed in other studies of EPI [12,29,46]. 

Aerobic GNB were found in 47% of EPIs using the Tsukayama classification, and 41.1% when considering infections that presented within 90 days of surgery. This is a high proportion, similar to percentages observed in other recent studies of PJI diagnosed in the first 3 months after surgery (39–42%) [11,47]. GNB have been considered an infrequent cause of PJI in the classic series, accounting for less than 10% of cases [41], although few studies have focused specifically on the etiology of PJI across the full range of infections, or were performed a long time ago, since when the microbiology of PJI is likely to have changed [8,14,41]. Nevertheless, the type of infection should always be taken into consideration. Accordingly, we observed that after the first month post-surgery, the percentage of non-hematogenous GNB PJIs steadily declined to 13.4% after the third month, which approaches the percentages found in the classic series. 

*C. acnes* is a major colonizer of the human skin that has recently emerged as a significant opportunistic pathogen able to cause implant-associated infections, typically with fewer clinical manifestations of infection than other bacteria [48]. This may explain why it was unexpectedly found more often in revision surgery (PIC) in our results than in other types of infection. 

As in previous studies, polymicrobial PJI was much more frequently found in EPI than in any other type [8]; the percentages of polymicrobial AHI and PIC cases were very low and the proportion of non-hematogenous polymicrobial PJIs decreased over time after the index surgery.

MDROs, which included mainly MRSA and multidrug-resistant GNB, were isolated in almost a quarter of EPIs, but rarely in other types of infection. Since no previous studies have studied the involvement of MDROs, and GNB especially, in terms of the category of infection, these results should be corroborated by other studies. Although the local epidemiology of MDROs will vary [49], our results suggest that, overall, they are more often isolated in EPIs. 

Based on our findings, some recommendations can be made for empirical antimicrobial treatment of PJIs. The regimen for a non-hematogenous PJI diagnosed in the first month following surgery should cover staphylococci, including methicillin-resistant staphylococci (with vancomycin or antibiotics with a similar spectrum, such as other glycopeptides or daptomycin) as well as *Enterobacteriaceae* and *P. aeruginosa* (with an antipseudomonal cephalosporin such as cefepime or ceftazidime). Overall, it seems that a broader-spectrum beta-lactam (such as piperacillin/tazobactam or a carbapenem) is not needed because of the low proportion of anaerobes and ESBL-*Enterobacteriaceae*. Likewise, in patients with non-hematogenous PJIs diagnosed in the second or third month after surgery, empirical regimens should also include methicillin-resistant staphylococci and *Enterobacteriaceae*, although coverage of *P. aeruginosa* may be unnecessary, and a third-generation non-antipseudomonal cephalosporin (ceftriaxone/cefotaxime) could be used instead of cefepime/ceftazidime. For non-hematogenous PJIs arising after the third month, empirical coverage of GNB (*Enterobacteriaceae* and *P. aeruginosa*) may not be required, also bearing in mind that these PJIs require removal of the prosthesis and the initial empirical therapy may not be so critical in this situation (although this issue has not so far been studied). For hematogenous infections, empirical treatment against *S. aureus*, streptococci, and *E. coli* (with a low percentage of multidrug-resistant *Enterobacteriaceae*) is recommended; coverage of MRSA should be considered even though this pathogen represents less than 10% of cases, because of the high mortality associated with MRSA bacteremia (which rises with inappropriate empirical therapy) [45]. Hence, a regimen including vancomycin (other glycopeptides/daptomycin) and ceftriaxone/cefotaxime could be envisaged. Empirical treatment may be needed for PIC until antibiogram data is available and coverage of methicillin-resistant staphylococci should be considered; it should also be borne in mind that *C. acnes* is usually susceptible to penicillin [43]. Despite the undoubted importance of rifampin and quinolones in the treatment of PJI, these agents are not recommended during the initial treatment phase of infection [2,6]. A preoperative synovial fluid culture is helpful for identification of the causative microorganism and determination of their antimicrobial susceptibility and, in addition, for informing the choice of postoperative antimicrobials. This can be very useful in chronic infections; although less so in acute infections since antimicrobial therapy is commonly started before the culture results are available. Nevertheless, a perioperative aspiration culture has shown a moderate average sensitivity of 68% [50].

There is at present no universally accepted classification of PJI [8,16]. The best classifications would be useful for therapeutic decision making, such as deciding on surgical treatment (debridement and implant retention or device removal) and starting appropriate empirical antimicrobial treatment. Following the Tsukayama scheme, EPI and AHI can potentially be cured with debridement and implant retention, while LCI requires prosthesis removal for cure. In the case of PIC, the surgical decision has already been made. Furthermore, each category of infection has a specific etiological pattern that facilitates tailored empirical treatment. LCIs presenting in months two to three and more than three months after surgery have different microbiological characteristics (mainly based on a significant difference in the incidence of CoNS and *Enterobacteriaceae*), which may allow empirical treatment to be refined. A new category that includes the two to three month postoperative period should therefore be considered. This new category is of further interest due to the possibility of curing PJIs without removal of the prosthesis during this period [6,51].

The limitations of our study are mainly related to its partial retrospective design, although it would be very difficult to collect such a large number of PJI cases with any other. The study assesses the microbial etiology of PJIs in our country and our results may not be generalizable to other countries, although other studies in different areas of the world have shown comparable results in many respects. Another strength of our study is its use of a standardized definition of MDRO [39], which enables comparisons of results with other centers. This definition of MDRO, however, has limitations in the context of PJI, since it does not include a definition of multidrug-resistant CoNS [39]. Nevertheless, the vast majority of clinically recovered CoNS isolates are methicillin-resistant [38], which determines the empirical treatment of PJIs. Our study also included all consecutive patients with a diagnosis of PJI, avoiding potential inclusion bias and guaranteeing that the various categories of infection were adequately represented. 

Our study provides detailed, comprehensive information about the microbial etiology of different categories of PJI. Notable differences in the causative microorganisms of these types of infection were found, which could be useful for optimizing empirical antimicrobial therapy of PJI and for improving the outcome of these infections. The Tsukayama classification is a useful guide for the treatment of PJI, although an extra category specific to non-hematogenous infections presenting in the second and third month after surgery should be considered.

## Figures and Tables

**Table 1:** Characteristics of patients with prosthetic joint infection diagnosed between 2003 and 2012.

Characteristic	No. of Cases (*n* = 2524)
Median age (IQR), years	74 (13)
Female gender	1508 (59.7)
Underlying conditions	
Any comorbid condition	1594 (63.3)
Diabetes mellitus	592 (23.5)
Heart disease	506 (20.1)
Chronic obstructive pulmonary disease	299 (11.9)
Cancer	231 (9.2)
Neurological disease	221 (8.8)
Chronic kidney disease	195 (7.7)
Systemic rheumatic (connective tissue) disease	175 (6.9)
Immunosuppressive treatment	168 (6.7)
Liver disease	164 (6.5)
Rheumatoid arthritis	129 (5.1)
Charlson score, median (IQR)	1 (2)
Index arthroplasty site	
Hip	1244 (49.3)
o Hemiarthroplasty	249 (9.9)
o Total arthroplasty	995 (39.5)
Knee	1219 (48.3)
Shoulder	46 (1.8)
Other	15 (0.6)
ASA score, median (IQR)	2 (1)
Indication for index arthroplasty*	
Primary joint replacement	1938 (77.2)
o Osteoarthritis	1264 (52.4)
o Fracture	417 (17.3)
o Avascular necrosis	51 (2.1)
o Rheumatoid arthritis	32 (1.3)
o Tumor	31 (1.3)
o Septic arthritis sequelae	12 (0.5)
o Other	43 (1.8)
Revision arthroplasty (prior joint arthroplasty)	573 (22.8)
o Aseptic loosening	292 (12.1)
o Infection	158 (6.6)
o Dislocation	32 (1.3)
o Periprosthetic fracture	25 (1)
o Implant failure or fracture	13 (0.5)
o Other	29 (1.2)

Unless stated otherwise, data refer to numbers (%) of patients with the indicated characteristic. ASA = American Society of Anesthesiologists; IQR = interquartile range. * Information on indication for index arthroplasty was not available for 112 (4.4%) procedures.

**Table 2:** Aerobic Gram-positive cocci involved in prosthetic joint infections using the four categories of the Tsukayama classification.

Microorganism or Microorganism Group	Early Postoperative Infections *n* = 839	Acute Hematogenous Infections *n* = 265	Late Chronic Infections *n* = 1052	Positive Intraoperative Cultures *n* = 126	*p*-Value
	Total no. (%)*				
*Staphylococcus* species	505 (60.2)	122 (46)	776 (**73.8**)	84 (**66.7**)	<0.001
Coagulase-negative Staphylococci (CoNS)	236 (28.1)	23 (8.7)	573 (**54.5**)	72 (**57.1**)	<0.001
o Staphylococcus epidermidis	130 (15.5)	11 (4.2)	355 (**33.7**)	36 (**28.6**)	<0.001
o Staphylococcus lugdunensis	2 (0.2)	6 (2.3)	31 (**2.9**)	4 (**3.2**)	<0.001
o Staphylococcus capitis	8 (1)	0 (0)	25 (**2**)	2 (**1.6**)	0.014
o Staphylococcus hominis	8 (1)	0 (0)	22 (**2.1**)	0 (0)	0.014
o Staphylococcus warneri	5 (0.6)	0 (0)	11 (**1**)	3 (**2.4**)	0.065
o Staphylococcus auricularis	2 (0.2)	0 (0)	12 (**1.1**)	1 (**0.8**)	0.048
o CoNS not identified to species level	89 (10.6)	6 (2.3)	168 (**16**)	23 (**23**)	<0.001
Staphylococcus aureus	299 (**35.6**)	104 (**39.2**)	224 (21.3)	12 (9.5)	<0.001
*Streptococcus* species	36 (4.3)	80 (**30.2**)	85 (8.1)	5 (4)	<0.001
Streptococcus agalactiae	8 (1)	28 (**10.9**)	28 (2.7)	0 (0)	<0.001
Viridans group streptococci not identified to species level	6 (0.7)	12 (**4.5**)	25 (2.4)	2 (1.6)	0.002
*Streptococcus mitis* group	8 (1)	5 (1.9)	16 (1.5)	2 (1.6)	0.632
Streptococcus anginosus group	3 (0.4)	8 (**3**)	12 (1.1)	1 (0.8)	0.005
Streptococcus pyogenes	10 (1.2)	5 (**1.9**)	1 (0.1)	0 (0)	0.003
Streptococcus pneumoniae	0 (0)	10 (**3.8**)	2 (0.2)	0 (0)	<0.001
Streptococcus dysgalactiae	2 (0.2)	7 (**2.6**)	1 (0.1)	0 (0)	<0.001
*Enterococcus* species	106 (**12.6**)	6 (2.3)	66 (6.3)	4 (3.2)	<0.001
Enterococcus faecalis	95 (**11.3**)	5 (1.9)	55 (5.2)	3 (2.4)	<0.001
Enterococcus faecium	7 (0.8)	1 (0.4)	5 (0.5)	0 (0.0)	0.584

* Percentages marked in bolded blue are statistically significant highest percentages in that row. Two percentages marked in bolded blue in the same row refer to the highest percentages (both are significantly higher than the other two percentages), but with no statistically significant differences between them.

**Table 3:** Microorganisms and group of microorganisms (other than aerobic Gram-positive cocci) involved in prosthetic joint infections according to the four categories of the Tsukayama classification.

Microorganism or Microorganism Group	Early Postoperative Infections *n* = 839	Acute Hematogenous Infections *n* = 265	Late Chronic Infections *n* = 1052	Positive Intraoperative Cultures *n* = 126	*p*-Value
	Total no. (%)*				
Aerobic Gram-negative bacilli	395 (**47.1**)	60 (22.6)	161 (15.3)	14 (11.1)	<0.001
Enterobacteriaceae	303 (**36.1**)	49 (18.5)	106 (10.1)	6 (4.8)	<0.001
o Escherichia coli	129 (**15.4**)	33 (**12.5**)	41 (3.9)	3 (2.4)	<0.001
o *Proteus* spp.	75 (**8.9**)	4 (1.5)	27 (2.6)	2 (1.6)	<0.001
o *Enterobacter* spp.	73 (**8.7**)	5 (1.9)	19 (1.8)	0 (0)	<0.001
o *Klebsiella* spp.	48 (**5.7**)	1 (0.4)	1 (0.4)	9 (0.9)	<0.001
o Morganella morganii	26 (**3.1**)	4 (1.5)	11 (1)	1 (0.8)	=0.009
o Serratia marcescens	13 (**1.5**)	0 (0)	6 (0.6)	0 (0)	=0.028
Non-fermenting Gram-negative bacilli	137 (**16.3**)	9 (3.4)	62 (5.9)	9 (7.1)	<0.001
o *Pseudomonas* spp.	128 (**15.3**)	8 (3)	59 (5.6)	6 (4.8)	<0.001
o *Acinetobacter* spp.	10 (**1.2**)	0 (0)	2 (0.2)	1 (0.8)	0.021
Aerobic Gram-positive bacilli	16 (1.9)	5 (1.9)	29 (2.8)	4 (3.2)	0.555
*Corynebacterium* species	16 (1.9)	1 (0.4)	29 (2.8)	4 (3.2)	0.087
o Corynebacterium striatum	9 (1.1)	0 (0)	7 (0.7)	1 (0.8)	0.321
o *Corynebacterium* spp. without identification to species level	3 (0.4)	1 (0.4)	14 (1.3)	2 (1.6)	0.081
Anaerobic Gram-positive bacilli	19 (2.3)	3 (1.1)	73 (**6.9**)**	22 (**17.3**)**	<0.001
*Cutibacterium* spp.	17 (2)	3 (1.1)	64 (**6.1**)**	19 (**15.1**)**	<0.001
Anaerobic Gram-positive cocci^†^	8 (1)	2 (0.8)	23 (**2.2**)	0 (0)	0.042
Anaerobic Gram-negative bacilli	12 (1.4)	1 (0.4)	8 (0.8)	0 (0)	0.182
*Bacteroides* group	10 (1.1)	1 (0.3)	5 (0.4)	0 (0)	0.145
*Mycobacterium* species	2 (0.2)	0 (0)	8 (0.8)	0 (0)	0.183
Fungi	8 (1.5)	4 (1.5)	16 (1.5)	2 (1.6)	0.723
*Candida* spp.	8 (1)	3 (1.1)	14 (1.3)	2 (1.6)	0.854
Multidrug-resistant organisms	201 (**24**)	29 (10.9)	82 (7.8)	6 (4.8)	<0.001
Methicillin-resistant *S. aureus*	92 (**11**)	22 (8.3)	58 (5.5)	5 (4)	<0.001
Multidrug-resistant Gram-negative bacilli	112 (**13.3**)	7 (2.6)	25 (2.4)	1 (0.8)	<0.001
*Extended*-spectrum beta-lactamase producing *Enterobacteriaceae*	36 (**4.3**)	2 (0.8)	4 (0.4)	0 (0.0)	<0.001
Ciprofloxacin-resistant Gram-negative bacilli	63 (**7.5**)	7 (2.6)	20 (1.9)	3 (2.4)	<0.001
Polymicrobial infections	230 (**27.4**)**	17 (6.3)	143 (**13.1**)**	7 (5.6)	<0.001

* Percentages marked in bolded blue are the highest statistically significant percentages in that row. Two percentages marked in bolded blue in the same row with no other marks, refer to the highest percentages (both are significantly higher than the other two percentages), but with no statistically significant differences between them. ** The two highest percentages in that row but with statistically significant differences between them. † *Finegoldia magna* 5, *Parvimonas micra* 5, *Peptostreptococcus anaerobius* 3, *Peptococcus niger* 4, *Peptostreptococcus* not identified to species level 15.

**Table 4:** Microorganisms and groups of organisms involved in non-hematogenous prosthetic joint infections according to time of infection after surgery (≤1 month, 2–3 months, 4–12 months, >12 months).

Microorganism or Microorganism Group	PJI within 1 Month after Surgery *n* = 844	PJI 2-3 Months after Surgery *n* = 243	PJI 4-12 Months after Surgery *n* = 277	PJI > 12 Months after Surgery *n* = 619	*p*-Value
	Total no. (%)*				
*Staphylococcus* species					
Coagulase-negative staphylococci	236 (28.2) **	107 (44) ** †	167 (**60.3**) †	348 (**56.2**)	<0.001
o Staphylococcus epidermidis	132 (15.6) **	68 (28) ** †	106 (**38.3**) †	203 (**32.8**)	<0.001
o Staphylococcus lugdunensis	2 (0.2) **	3 (1.3) **	10 (**3.6**)	22 (**3.6**)	<0.001
Staphylococcus aureus	301 (**35.7**)	60 (24.7)	60 (21.7)	108 (17.4)	<0.001
*Streptococcus* species	36 (4.3) **	25 (**10.3**) **	14 (5.1)	49 (7.9)	<0.001
o Streptococcus agalactiae	8 (0.9) **	11 (**4.5**) **	6 (2.2)	10 (1.6)	0.003
o Viridans group streptococci not identified to species level	6 (0.7) **	7 (**2.9**) **	2 (0.7) †	18 (**2.9**) †	0.003
*Enterococcus* species	106 (**12.6**)	23 (9.5)	15 (5.4)	32 (5.4)	<0.001
Aerobic Gram-negative bacilli	396 (**46.9**) **	50 (20.6) ** †	37 (13.4) †	37 (13.4)	<0.001
Enterobacteriaceae	303 (**35.9**) **	37 (15.2) ** †	25 (9) †	48 (7.8)	<0.001
o Escherichia coli	129 (**15.3**)	12 (4.9)	10 (3.6)	21 (3.4)	<0.001
o *Proteus* spp.	75 (**8.9**)	7 (2.9)	7 (2.5)	14 (2.3)	<0.001
o *Enterobacter* spp.	73 (**8.6**) **	11 (4.5) ** †	2 (0.7) †	6 (1)	<0.001
o *Klebsiella* spp.	48 (**5.7**)	2 (0.8)	4 (1.4)	3 (0.5	<0.001
Non-fermenting Gram-negative bacilli	138 (**16.4**) **	16 (6.6) **	11 (4)	41 (6.6)	<0.001
o *Pseudomonas* spp.	128 (**15.2**) **	17 (7) **	11 (4)	35 (5.7)	<0.001
Aerobic Gram-positive bacilli	16 (1.9)	3 (1.2)	7 (2.5)	23 (3.7)	0.083
Anaerobic Gram-positive bacilli	19 (2.3) **	14 (5.7) **	16 (5.8)	61 (9.7)	<0.001
*Cutibacterium* spp.	17 (2) **	12 (4.9) **	16 (5.8)	51 (8.2)	<0.001
Anaerobic Gram-positive cocci	8 (0.9)	4 (1.6)	5 (1.8)	13 (2.1)	0.330
Anaerobic Gram-negative bacilli	12 (1.4)	2 (0.8)	1 (0.4)	5 (0.8)	0.409
*Mycobacterium* species	2 (0.2)	1 (0.9)	4 (1.4)	2 (0.3)	0.068
Fungi	8 (0.9)	3 (1.2)	3 (1.1)	12 (1.9)	0.418
Multidrug-resistant organisms	202 (**23.9**)	20 (8.2)	20 (7.2)	43 (6.9)	<0.001
Methicillin-resistant *S. aureus*	93 (**11**)	14 (5.8)	14 (5.1)	30 (4.8)	<0.001
Multidrug-resistant Gram-negative bacilli	112 (**13.3**)	7 (2.5)	5 (1.8)	14 (2.3)	<0.001
Extended-spectrum beta-lactamases producing *Enterobacteriaceae*	36 (**4.3**)	1 (0.4)	2 (0.7)	1 (0.3)	<0.001
Ciprofloxacin-resistant Gram-negative bacilli	84 (**10**)	5 (2.1)	4 (1.4)	11 (1.8)	<0.001
Polymicrobial infections	230 (**27.2**)	36 (14.7)	32 (11.1)	77 (11.1)	<0.001

* Percentages marked in bolded blue are the highest statistically significant percentages in that row. Two percentages marked in bolded blue in the same row with no other indication refer to the highest percentages (both are significantly higher than the other two percentages) with no statistically significant differences between them. ** and † indicate adjacent percentages with statistically significant differences between them in the same row.
